# Outcomes of patients with heart failure in Türkiye

**DOI:** 10.55730/1300-0144.5935

**Published:** 2024-06-14

**Authors:** Anıl ŞAHİN, Mehmet Birhan YILMAZ, Ahmet ÇELİK, İnci Tuğçe ÇÖLLÜOĞLU, Dilek URAL, Lale Dinç ASARCIKLI, Sanem NALBANTGİL, Emre DEMİR, Yüksel ÇAVUŞOĞLU, Selda MURAT, Emine Arzu KANIK, Naim ATA, Mustafa Mahir ÜLGÜ, Şuayip BİRİNCİ

**Affiliations:** 1 Department of Cardiology, Faculty of Medicine, Sivas Cumhuriyet University, Sivas, Turkiye; 2 Department of Cardiology, Faculty of Medicine, Dokuz Eylül University, İzmir, Turkiye; 3 Department of Cardiology, Faculty of Medicine, Mersin University, Mersin, Turkiye; 4 Department of Cardiology, Faculty of Medicine, Karabük University, Karabük, Turkiye; 5 Department of Cardiology, Faculty of Medicine, Koç University, İstanbul, Turkiye; 6 Department of Cardiology, Faculty of Medicine, Health Sciences University, İstanbul, Turkiye; 7 Department of Cardiology, Faculty of Medicine, Ege University, İzmir, Turkiye; 8 Department of Cardiology, Faculty of Medicine, Eskişehir Osmangazi University, Eskişehir, Turkiye; 9 Department of Biostatistics, Faculty of Medicine, Mersin University, Mersin, Turkiye; 10 General Directorate of Health Information Systems, Ministry of Health, Ankara, Turkiye; 11 Ministry of Health, Ankara, Turkiye

**Keywords:** Heart failure, sex, length of stay, mortality

## Abstract

**Background/aim:**

Despite Türkiye’s relatively young population, there is an emerging trend of earlier diagnoses of chronic diseases, including heart failure (HF). This study aims to shed light on survival rates, potential influences of guideline-directed therapies, and sex-based differences necessitating personalized management in HF.

**Materials and methods:**

We conducted a nationwide retrospective cohort analysis of 2,722,151 patients with HF using deidentified data from the Turkish Ministry of Health’s national electronic database. That cohort included 2,701,099 adult patients with HF. Adult patients were divided into two groups based on their outcomes as those who were deceased and those who survived and were then compared. Multivariate regression analysis was conducted to identify variables predicting mortality. The patients’ hospital admissions and length of hospital stay were analyzed based on survival status and age.

**Results:**

Out of 2,722,151 HF patients, the overall mortality rate was 33.7%, with a difference observed according to sex (32.5% in female patients, 35.0% in male patients). Survival rates at 1, 5, and 7 years after the HF diagnosis were detailed. Deceased HF patients had more comorbidities, higher natriuretic peptides, and lower glomerular filtration rates. Hospitalization patterns varied, with 41% experiencing no hospitalization. The average length of hospital stay in 2022 was 6 days, with sex- and age-specific disparities.

**Conclusion:**

The survival rate of HF in Türkiye is similar to world data. The survival of female patients is better than that of male patients. Increased survival rates can likely be attributed to the widespread use of guideline-directed therapies. Finally, high healthcare utilization is observed, especially in emergency situations.

## 1. Introduction

Heart failure (HF) is a chronic noncommunicable disease that results from structural and functional abnormalities of the heart yielding significant morbidity and mortality [1]. Owing to changes in lifestyle brought on by aging populations, diabetes mellitus (DM), hypertension (HT), obesity, and increased rates of survival following myocardial infarction (MI), it is anticipated that the prevalence of HF and the accompanying health and financial burdens will increase worldwide [2]. The quality of life is drastically reduced by this potentially fatal illness, which also continues to be a leading cause of death for those who are affected. HF is still linked to high rates of death and readmission despite tremendous advances in pharmacological and nonpharmacological therapies over the past 20 years. This has resulted in massive healthcare expenses [3,4].

Despite having a younger population than European nations, Türkiye has been noted for experiencing higher rates of various chronic noncommunicable diseases, such as HF, earlier in life [5,6]. Since HF can be fatal, there is a dearth of data regarding the outcomes of HF patients in the entire nation. The TRends-HF study, aiming to evaluate the entire population of Türkiye to provide an overview of the epidemiology and outcomes of HF in the country and data regarding adult HF patients, was recently published, although outcome data on specific age groups were not provided [7,8]. The present study focuses specifically on major outcomes, including hospitalization and death, in adult HF patients in general and then among distinct age groups (0–19 versus 20–49 versus ≥50 years) of Turkish HF patients together with sex-based analysis.

## 2. Materials and methods

This study was based on anonymized data obtained from the Turkish Ministry of Health’s national electronic database. There are seven geographical regions registered in the database, accounting for a total of nearly 85 million people. The data of 2,722,151 Turkish residents who were diagnosed with HF, sought treatment, and were monitored in all public and private healthcare facilities in the nation between 1 January 2016 and 31 December 2022 were included in this study with permission from the Turkish Ministry of Health. Protocol number 95741342-020 was used to perform this retrospective cohort study upon the authorization of the Ministry of Health. The study was conducted in accordance with the Declaration of Helsinki. A document was created using the STROBE guidelines and checklist for cohort studies.

The patients’ diagnoses were systematically coded according to the 10th revision of the International Classification of Diseases (ICD-10), a standardized coding system implemented in Türkiye since the year 2016. HF cases were specifically identified through the application of ICD-10 codes, namely I50.0 (congestive HF), I50.1 (left ventricular dysfunction), I50.9 (unspecified HF), I11.0 (hypertensive heart disease with congestive HF), I13.0 (hypertensive heart and chronic kidney disease with congestive HF), I13.2 (hypertensive heart and chronic kidney disease with congestive HF and renal failure), and I42.0 (dilated cardiomyopathy). The study incorporated an array of diverse variables, encompassing demographic attributes such as age and sex alongside pertinent clinical details such as the date of initial diagnosis, the healthcare institution responsible for the diagnosis, the geographical distribution of HF patients across cities and regions, and the categorization of HF patients based on hospitalization dates. Additional variables included the nature of hospital admissions, duration of hospital stay, date of death, and the duration of time from diagnosis to death. These datasets were meticulously gathered from the Ministry of Health’s national electronic database. Moreover, mortality information was extracted from the Death Notification System maintained by the General Directorate of Health Information Systems. Patients aged 18 and older were subjected to a separate analysis after forming two groups based on outcomes, including deceased patients and those who survived. Comparative assessments were conducted according to their baseline demographic features, HF treatment modalities, and laboratory characteristics.

Cross-tables were generated for categorical variables, expressing values as case numbers and percentages. Age and laboratory parameters at the time of the initial HF diagnosis were described using median and interquartile range [25th–75th] values. For survival analyses, the life table method was employed for overall survival. Regression analysis was performed on the statistically significant parameters obtained from the univariate analysis and independent predictors of all-cause mortality were investigated. Statistical analyses were performed using IBM SPSS Statistics 29.0 (IBM Corp, Armonk, NY, USA) and E-PICOS AI (MedicReS, New York, NY, USA).

## 3. Results

**Figure 1:**
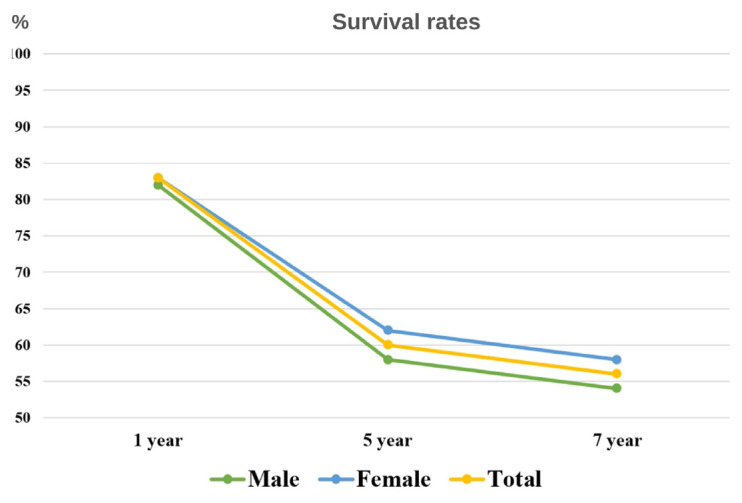
Sex-based survival rates in heart failure cases.

Out of a total cohort of 2,722,151 HF patients, the all-cause mortality analysis covering the period from 1 January 2016 to 31 December 2022 showed an overall mortality rate of 33.7%, with 918,514 fatalities, as reported previously [5]. A substantial difference was observed in terms of sex, as 32.5% of female patients (458,108 of 1,407,927) and 35% of male patients (460,406 out of 1,314,224) died during this period. Survival rates were determined at 1, 5, and 7 years after the diagnosis of HF. In contrast to the male population, which had survival rates of 82.1% (95% CI: 82.0–82.2), 58.2% (95% CI: 58.1–58.3), and 54.2% (95% CI: 54.0–54.3), the female population had survival rates of 83.3% (95% CI: 83.2–83.3), 61.5% (95% CI: 61.4–61.6), and 57.7% (95% CI: 57.6–57.8) (Figure 1).

Within the adult patient subgroup, an in-depth examination revealed that those who died had a median age of 77 (69–83) years, while survivors had a median age of 66 (58–74) years. Predominant comorbidities among deceased HF patients included chronic obstructive pulmonary disease (COPD) in 56.9%, atrial fibrillation (AF) in 44.1%, DM in 44.0%, and anemia in 43.5% (Table 1). Additionally, deceased adults exhibited elevated levels of natriuretic peptides coupled with diminished estimated glomerular filtration rate (eGFR) and hemoglobin levels (Table 1). Noteworthy differences in the utilization of HF treatments were observed, with the surviving cohort demonstrating higher percentages of beta-blocker (BB), renin-angiotensin system inhibitor (RASi), and sodium-glucose cotransporter-2 inhibitor (SGLT-2i) use.

**Figure 2:**
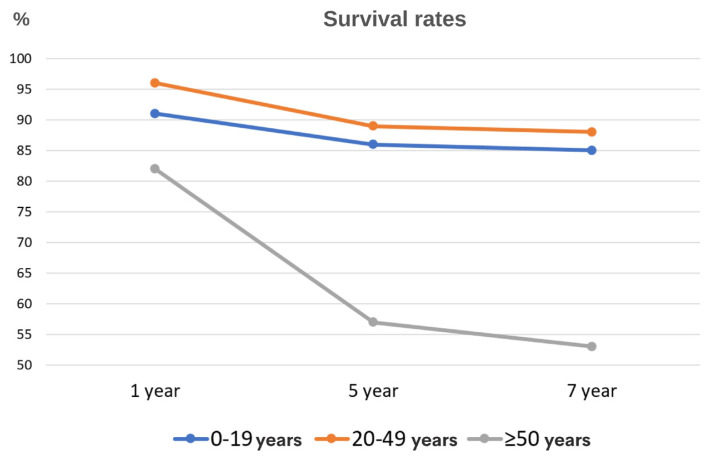
Survival based on selected age groups.

Survival rate analyses across distinct age groups (0–19, 20–49, and ≥50 years) revealed nuanced trends. At the 12th month, survival rates were 91% for the age group of 0–19 years, 96% for those aged 20–49 years, and 82% for those aged ≥50 years. By the 60th month, survival rates were 86%, 89%, and 57% for the respective age groups, further declining to 85%, 88%, and 53% by the 84th month (Figure 2).

**Figure 3:**
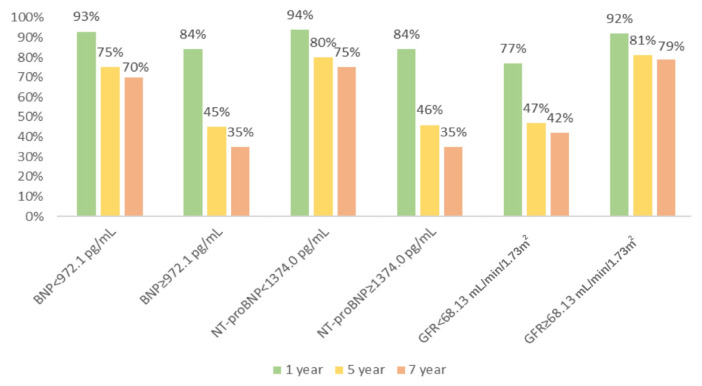
Survival rates according to median laboratory parameters in heart failure patients.

Sex-stratified survival rates coupled with median laboratory parameter values, as depicted in Figure 3, underscored the inverse correlation between both B-type natriuretic peptide (BNP) and N-terminal pro B-type natriuretic peptide (NT-proBNP) levels and survival rates, while eGFR exhibited a positive correlation. Predictors of all-cause mortality by sex are comprehensively presented in Table 2.

Examining healthcare burden, individuals who died of HF in 2016–2022 had higher average rates of hospitalizations, emergency room visits per person compared to survivors. The average number of hospitalizations due to HF was 2.33 for the deceased and 1.46 for survivors. However, the average number of cardiology outpatient clinic visits was 10.02 for deceased patients and 14.06 for survivors (Table 3).

**Figure 4:**
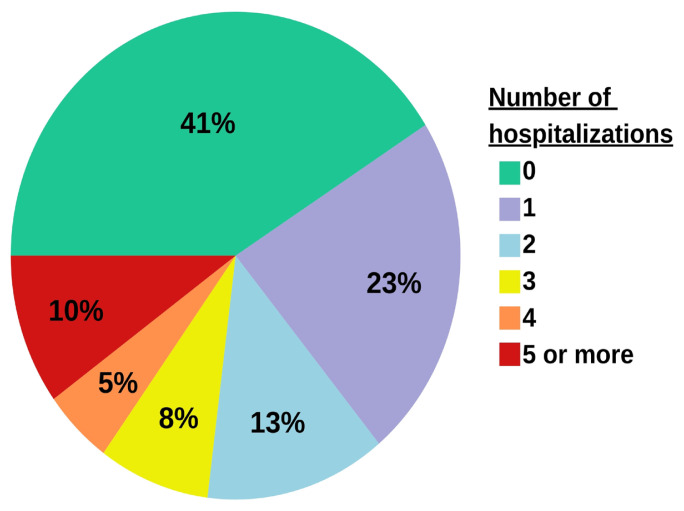
Number of hospitalizations throughout the follow-up period.

Hospitalization patterns for patients diagnosed with HF between 2016 and 2022 indicated that 41% experienced no hospitalizations, while 10% were admitted five times or more (Figure 4). Additionally, Table 4 presents hospitalization and admission data by age groups, revealing a positive correlation between average hospitalization rates per person and patient age. The age group of 20–49 years had the highest average numbers of emergency room visits (27.83) and cardiology outpatient clinic visits (15.01).

A subsequent analysis spanning from 1 January 2022 to 31 December 2022 revealed that 36.7% of surviving HF patients had a history of hospitalization within that 1 year of follow-up. The median hospital stay for HF patients in 2022 was 6 (2–13) days, with similar durations for male and female patients. Notably, age-specific analyses indicated prolonged hospitalization durations in the age groups of 0–9 years and ≥80 years (Table 5).

## 4. Discussion

Based on a national database, our study includes data covering the entire population of Türkiye as well as a roughly 7-year follow-up duration. As a result, it offers distinct information on how HF affects survival. Regional differences may be present in HF mortality rates. From this perspective, our research will help clarify and potentially change how HF is managed in this country.

In Türkiye, the 1-year survival rate for HF patients is 83%, with a corresponding 5-year survival rate of 60%. These rates are in line with epidemiological observations from Western countries [9–11]. On a global scale, the mortality outcomes for patients discharged following acute HF presentations reveal a 20% mortality rate within 1 year after discharge. This rate exhibits regional variations, ranging from 16% in East Europe to 22% in the Eastern Mediterranean, Africa, and Latin America [12,13]. Regarding ambulatory chronic HF patients from diverse geographic regions, a prospective cohort study reported noteworthy 1-year mortality rates, notably 34% in Africa and 23% in India, with the lowest rate of 9% observed in the Middle East [14]. A parallel examination of European HF patients after hospitalization indicated a 1-year survival rate after hospitalization averaging 70%, with a 5-year survival rate averaging 45% [15]. The study by Dural et al., scrutinizing age-adjusted mortality patterns among HF patients in Türkiye, documented a discernible decline in mortality rates, particularly evident from the year 2016 onwards [16]. This decline may be attributed to the increased adoption of guideline-directed therapy, most notably with the introduction of molecules such as ARNI and SGLT-2i. Our investigation further corroborates this trend, revealing elevated usage rates of BB, RASi, and SGLT-2i treatments in the cohort of surviving patients compared to their deceased counterparts. On the contrary, furosemide use appears to have been more common in the deceased patient group. This shows that diuretic treatment used to control symptoms is related to functional capacity and that outcomes are poorer in the group of patients who are more symptomatic due to HF.

It is helpful to draw parallels between these national data and the SELFIE-TR study, which systematically examined mortality in both acute and chronic HF patients across all ejection fraction groups [17]. While our study offers a comprehensive dataset representing the entire population of Türkiye, the congruence of our findings with those of the SELFIE-TR study underscores the robustness and generalizability of the observed trends across distinct cohorts and settings. Similar to the findings of the SELFIE-TR study, our study also revealed a higher median age in the group of deceased patients. In our analysis, which included adult patients, a higher mortality burden was observed in the group of patients who died. Previous studies have consistently demonstrated associations between worse prognosis and comorbidities such as prior MI, COPD, AF, and anemia [17–19].

Upon scrutinizing the mortality rates of patients with HF based on sex, a discernible trend emerged, indicating a lower all-cause mortality rate among female HF patients compared to their male counterparts. Particularly noteworthy is the observation that the 84-month survival rate was better among women compared to men. These findings align with historical data, such as the Framingham registry study [20], consistently illustrating a more favorable prognosis for HF in women. This sex-based divergence in outcomes may be ascribed to the improved prognosis associated with the HFpEF phenotype, a presentation more prevalent in women.

Noteworthy sex-specific variations exist in factors predicting HF mortality. Within the adult age group, advanced age and COPD exert a more pronounced impact on mortality in men, whereas the presence of a history of MI and DM in women may contribute to an increased risk of all-cause mortality. The subdivision of the population into age groups revealed that the group aged 50 and above had the poorest survival rates. A study conducted by Shah et al. supports this observation, as it indicated that in HF patients, and particularly those aged 65 and above, life expectancy experiences a rapid decline with increasing age [21]. Notably, our population exhibited distinctive characteristics, with the age of HF diagnosis and the onset of declining life expectancy found to be approximately a decade earlier than corresponding values in Western countries.

In addition to the progressive course of the disease in HF patients, there are many factors that affect outcomes, such as comorbidities and treatment use [10,17]. It is known that survival decreases with increasing comorbidity burden in HF patients [13]. In our study, ischemic heart diseases, COPD, AF, anemia, and cerebrovascular diseases were more common in the deceased patient group. This is in line with the findings of previous studies, which have indicated that as the comorbidity burden increases in HF patients, the outcomes tend to worsen. We examined independent factors that can predict outcomes differently according to sex. In particular, increasing age and the presence of DM, COPD, or ischemic etiology were shown to increase all-cause mortality in both sexes. However, there are differences according to sex in the effects of comorbidities on outcomes. For example, the presence of DM increases the risk of all-cause death by 32% in women, while this rate was found to be 10% in men. It was also observed that advancing age has a higher contribution to mortality, especially in men. Additionally, the positive effects of traditional disease-modifying HF treatments on outcomes are consistent in both sexes. In this respect, it is important to apply appropriate diagnosis and follow-up for comorbidities, regardless of sex, in addition to optimal HF treatment under guideline guidance in HF patients.

Within the scope of this analysis, we also reviewed hospital admissions and inpatient stays among HF patients. Our analysis revealed a noteworthy pattern, indicating that the incidence of emergency room admissions for patients with HF in Türkiye surpasses outpatient visits to cardiology clinics. Conventionally, it is presumed that hospital admissions in the community predominantly transpire through emergency services. However, this prevailing trend potentially amplifies the patient burden on emergency departments, introducing the risk of delayed access to appropriate diagnostic and therapeutic interventions for individuals with HF.

Moreover, a conspicuous sex-based distinction surfaced as we observed that male HF patients exhibited a higher frequency of seeking medical attention and a correspondingly higher rate of hospitalizations compared to their female counterparts. This sex-specific variation may be ascribed to the higher prevalence of atherosclerotic cardiovascular disease and HFrEF in male patients. Additional considerations include the influence of regional or cultural factors contributing to this observed sex disparity.

Expanding our analysis to encompass hospital admission numbers stratified by age groups, a salient finding emerged, highlighting that the most frequent admissions occurred within the age group of 20–49 years. Furthermore, a positive correlation was discerned between advanced age and escalating numbers of hospitalizations. It is noteworthy that our study revealed that 41% of HF patients had never undergone hospitalization, shedding light on the diverse clinical trajectories within this patient cohort. This multifaceted analysis contributes valuable insight on the healthcare landscape for HF patients, underscoring the need for nuanced strategies to address the varied patterns of healthcare utilization observed within this population.

Our analysis for 2022 showed that the average length of hospital stay for HF patients in Türkiye was 6 days. Notably, this duration exhibited sex-based disparities, with male patients experiencing longer hospital stays compared to female patients. In contrast to Western countries, where the average length of hospital stay for HF patients is reported to be 8.5 days [22], our findings suggest a potential challenge in achieving optimal decongestion in patients within our healthcare system. Prolonged hospitalizations in male patients may be indicative of greater clinical complexity or delayed response to therapeutic interventions.

Furthermore, an interesting trend was identified among HF patients aged 0–19, who tended to have an extended duration of hospital stay. In comparison to global benchmarks, the length of hospitalization for HF patients in Türkiye over the course of the considered year appears to be comparatively lower. It is crucial to underscore the significance of optimal treatment and complete decongestion before discharging HF patients, as premature discharge is associated with increased mortality rates. Therefore, the observed pattern in this country, potentially reflecting suboptimal decongestion, raises concerns about the risk of heightened readmissions and adverse outcomes in this patient population. Addressing these disparities and enhancing the quality of care, particularly with regard to achieving optimal decongestion, is imperative to mitigate adverse outcomes and improve the overall prognosis for patients with HF in our healthcare setting.

Natriuretic peptides are biomarkers that can help diagnose, treat, and determine the prognosis of patients with HF [23]. Many studies have been conducted on their short- and long-term predictability, particularly regarding their prognostic benefits [24,25]. However, the data on this topic are still contradictory. It is important to note that natriuretic peptide levels can be influenced by various cardiac and noncardiac conditions besides HF, limiting their prognostic usefulness. In our study, we identified the median values of natriuretic peptides in a large population of HF patients. Moreover, we observed differences in survival rates among patients with values above and below these median levels.

This study has several strengths. First of all, the fact that it is based on a comprehensive national dataset including approximately 85 million people in Türkiye and covering a 7-year period (2016–2022) increases the general validity of the results and allows for more reliable statistical analyses. Additionally, the study includes various variables such as demographic characteristics, clinical data, hospitalizations, emergency department visits, and laboratory values, making it possible to comprehensively analyze the condition of patients with HF. Analyses by sexes and age groups revealed the effects of HF on different demographic groups, emphasizing the importance of personalized treatment approaches. Determining healthcare utilization can enable the identification of burdens on the healthcare system and potential areas for improvement. Additionally, comparisons of the outcomes of HF patients in Türkiye with international data revealed how the situation across the country compares to the global context.

Our study also has limitations that are worth mentioning. The data were collected through a retrospective analysis of an electronic dataset; hence, the study has some potential biases related to the selection process and other variables that may impact the outcomes. It is important to acknowledge that the study design emphasizes associations rather than causation, implying that our ability to establish causal relationships or draw definitive conclusions may be limited due to the retrospective nature of the data. One of the primary limitations of the study is the incomplete availability of transthoracic echocardiography data, particularly regarding left ventricular ejection fraction (LVEF), at the time of the index diagnosis. Hence, it was not possible to define HF phenotypes. Furthermore, since the diagnosis of HF was made based on ICD-10 codes and the ICD-10 system does not make a distinction based on ejection fraction, we were unable to classify our patients according to LVEF. Another limitation was associated with the transition to the national database after 2016, resulting in a limited timeframe for the available data. This transition has implications for the generalizability of our findings to a broader time period. It is important to consider these limitations while interpreting the results of our study.

## 5. Conclusion

Our comprehensive national database study conducted in Türkiye has yielded valuable insights into the epidemiology and outcomes of HF over a span of 7 years. Notwithstanding the country’s relatively younger demographic profile, the diagnosis of chronic conditions such as HF is occurring at earlier ages. The enhanced survival rates observed in our study could be attributed to the widespread utilization of guideline-directed therapies. Furthermore, our investigation has discerned notable sex-based differences in the HF landscape, underscoring the imperative for personalized management strategies. A key finding from our study pertains to the high healthcare utilization observed, particularly in emergency settings, indicating potential areas for improvement in the healthcare delivery system. Despite the limitations inherent in any study, our findings constitute valuable information that can serve as a foundation for shaping healthcare policies. These insights are particularly pertinent in addressing the dynamic and evolving landscape of HF in Türkiye, providing a basis for informed decision-making and strategic planning to enhance the overall management and outcomes for individuals with HF in this country.

## Figures and Tables

**Table 1:** Comparison of adult heart failure patients.

Variables	Survivors n = 1,785,202	Deceased n = 915,897	p-value
Female, n (%)	941,165 (52.7)	456,890 (49.9)	<0.001
Age (years)	66 (58–74)	77 (69–83)	<0.001
Prior MI, n (%)	362,140 (20.3)	229,766 (25.1)	<0.001
Diabetes mellitus, n (%)	827,272 (46.3)	402,561 (44.0)	<0.001
Atrial fibrillation, n (%)	606,112 (34.0)	403,541 (44.1)	<0.001
COPD, n (%)	664,117 (37.2)	521,381 (56.9)	<0.001
Anemia, n (%)	699,333 (39.2)	398,635 (43.5)	<0.001
Pulmonary embolism, n (%)	113,987 (6.4)	97,432 (10.6)	<0.001
Ischemic stroke, n (%)	92,902 (5.2)	85,196 (9.3)	<0.001
Hemorrhagic stroke, n (%)	11,333 (0.6)	11,262 (1.2)	<0.001
Beta-blockers, n (%)	1,525,281 (85.4)	739,786 (80.8)	<0.001
RASi, n (%)	971,879 (54.4)	455,772 (49.8)	<0.001
MRA, n (%)	646,734 (36.2)	404,970 (44.2)	<0.001
SGLT-2i, n (%)	261,219 (14.6)	37,362 (4.1)	<0.001
Furosemide, n (%)	984,062 (55.1)	680,089 (74.3)	<0.001
Hemoglobin (g/dL)	12.1 (9.6–13.8)	10.7 (8.6–12.6)	<0.001
BNP (pg/mL)	695 (185–2354)	2251 (628–5000)	<0.001
NT-proBNP (pg/mL)	993 (264–3047)	3333 (976–9706)	<0.001
eGFR (CKD-EPI)	79.8 (60.0–93.9)	63.0 (42.2–83.7)	<0.001

Categorical variables are presented as n (%), continuous variables are presented as median (IQR 25–75th). BNP, b-type natriuretic peptide; COPD, chronic obstructive pulmonary disease; eGFR, estimated glomerular filtration rate; MI, myocardial infarction; MRA, mineralocorticoid receptor antagonist; NT-proBNP, N-terminal pro b-type natriuretic peptide; RASi, renin angiotensin system inhibitor; SGLT-2i, sodium/glucose cotransporter 2 inhibitor.

**Table 2:** All-cause mortality predictors in each sex.

Variables	Female		Male	
	OR (95% CI)	p-value	OR (95% CI)	p-value
Age	1.07 (1.07–1.07)	<0.001	1.54 (1.53–1.54)	<0.001
Prior MI	1.36 (1.35–1.37)	<0.001	1.23 (1.22–1.24)	<0.001
Diabetes mellitus	1.32 (1.26–1.39)	<0.001	1.10 (1.09–1.11)	<0.001
COPD	1.24 (1.23–1.25)	<0.001	1.41 (1.40–1.42)	<0.001
Pulmonary embolism	1.15 (1.14–1.16)	<0.001	1.17 (1.16–1.18)	<0.001
Ischemic stroke	1.23 (1.22–1.25)	<0.001	1.16 (1.15–1.17)	<0.001
Hemorrhagic stroke	1.22 (1.19–1.25)	<0.001	1.21 (1.18–1.24)	<0.001
Beta-blockers	0.80 (0.79–0.81)	<0.001	0.72 (0.71–0.73)	<0.001
RASi	0.82 (0.82–0.83)	<0.001	0.80 (0.79–0.80)	<0.001
MRA	1.08 (1.08–1.09)	<0.001	1.13 (1.12–1.14)	<0.001
SGLT-2i	0.44 (0.43–0.44)	<0.001	0.40 (0.39–0.40)	<0.001

COPD, chronic obstructive pulmonary disease; MI, myocardial infarction; MRA, mineralocorticoid receptor antagonist; RASi, renin angiotensin system inhibitor; SGLT-2i, sodium/glucose cotransporter 2 inhibitor.

**Table 3:** Overall healthcare burden of heart failure.

	Average number of emergency service admissions	Average number of cardiology outpatient visits	Total number of hospitalizations due to HF
Survivors (n = 1,803,637)	21.90	14.06	1.46
Deceased (n = 918,514)	23.36	10.02	2.33
Total (n = 2,722,151)	22.39	12.76	1.75

**Table 4:** Healthcare burden in preset age groups.

Age groups (years)	Average number of emergency service admissions	Average number of cardiology outpatient visits	Total number of hospitalizations due to HF
0–19 (n = 23,443)	21.87	2.17	0.40
20–49 (n = 201,773)	27.83	15.01	1.68
≥50 (n = 2,496,935)	21.96	12.68	1.77

**Table 5:** Length of hospital stays according to sex and decades of life.

		Sex		Age groups (years)									
	Total	Male	Female	0–9	10–19	20–29	30–39	40–49	50–59	60–69	70–79	80–89	≥90
Length of hospital stays (days)	6 (2–13)	6 (2–14)	6 (2–13)	7 (2–16)	5 (1–14)	3 (1–12)	3 (1–11)	4 (1–10)	4 (1–12)	6 (2–13)	6 (2–14)	7 (3–15)	7 (3–15)
